# Proteomic Profiling of Mouse Epididymosomes Reveals their Contributions to Post-testicular Sperm Maturation[Fn FN1]

**DOI:** 10.1074/mcp.RA118.000946

**Published:** 2018-09-13

**Authors:** Brett Nixon, Geoffry N. De Iuliis, Hanah M. Hart, Wei Zhou, Andrea Mathe, Ilana R. Bernstein, Amanda L. Anderson, Simone J. Stanger, David A. Skerrett-Byrne, M. Fairuz B. Jamaluddin, Juhura G. Almazi, Elizabeth G. Bromfield, Martin R. Larsen, Matthew D. Dun

**Affiliations:** From the ‡Priority Research Centre for Reproductive Science, School of Environmental and Life Sciences, Discipline of Biological Sciences, The University of Newcastle, University Drive, Callaghan, NSW 2308, Australia;; §Department of Biochemistry and Molecular Biology, University of Southern Denmark, Campusvej 55, DK-5230 Odense M, Denmark;; ¶School of Biomedical Sciences and Pharmacy, Faculty of Health and Medicine, The University of Newcastle, Callaghan, NSW 2308, Australia;; ‖Hunter Medical Research Institute, Cancer Research Program, New Lambton Heights, NSW 2305, Australia

**Keywords:** Exosomes, Quantification, 2-D Gel Electrophoresis, Cell biology*, Developmental biology*, Mass Spectrometry, Mouse models, epididymis, epididymosome, sperm, sperm

## Abstract

The functional maturation of spermatozoa that is necessary to achieve fertilization occurs as these cells transit through the epididymis, a highly specialized region of the male reproductive tract. A defining feature of this maturation process is that it occurs in the complete absence of nuclear gene transcription or *de novo*, protein translation in the spermatozoa. Rather, it is driven by sequential interactions between spermatozoa and the complex external milieu in which they are bathed within lumen of the epididymal tubule. A feature of this dynamic microenvironment are epididymosomes, small membrane encapsulated vesicles that are secreted from the epididymal soma. Herein, we report comparative proteomic profiling of epididymosomes isolated from different segments of the mouse epididymis using multiplexed tandem mass tag (TMT) based quantification coupled with high resolution LC-MS/MS. A total of 1640 epididymosome proteins were identified and quantified via this proteomic method. Notably, this analysis revealed pronounced segment-to-segment variation in the encapsulated epididymosome proteome. Thus, 146 proteins were identified as being differentially accumulated between caput and corpus epididymosomes, and a further 344 were differentially accumulated between corpus and cauda epididymosomes (*i.e.*, fold change of ≤ −1.5 or ≥ 1.5; *p*, < 0.05). Application of gene ontology annotation revealed a substantial portion of the epididymosome proteins mapped to the cellular component of extracellular exosome and to the biological processes of transport, oxidation-reduction, and metabolism. Additional annotation of the subset of epididymosome proteins that have not previously been identified in exosomes revealed enrichment of categories associated with the acquisition of sperm function (*e.g.*, fertilization and binding to the zona pellucida). In tandem with our demonstration that epididymosomes are able to convey protein cargo to the head of maturing spermatozoa, these data emphasize the fundamental importance of epididymosomes as key elements of the epididymal microenvironment responsible for coordinating post-testicular sperm maturation.

## Abstract (precis)

The proteomic composition of extracellular vesicles (epididymosomes) secreted by the mouse epididymis has been determined by applying multiplexed tandem mass tag based quantification coupled with high resolution LC-MS/MS. This analysis confirmed that epididymosomes encapsulate an extremely rich and diverse proteomic cargo, which is commensurate with their putative role in coordinating the post-testicular maturation and storage of spermatozoa.

## Graphical Abstract

**Figure:**
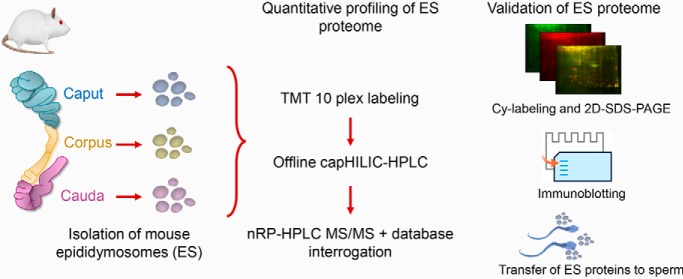

**Highlights**

- Comparative proteomics of extracellular vesicles isolated from the mouse epididymis.
- Epididymosome proteome displays pronounced segment-to-segment variation.
- Epididymosomes deliver protein cargo to the sperm head.
- Mechanistic insights into role of epididymosomes in sperm maturation and storage.

Mammalian spermatozoa acquire the potential to fertilize an ovum as they navigate the epididymis, an exceptionally long convoluted tubule that connects the testis to the vas deferens. This maturation process encompasses a suite of cellular modifications that endow spermatozoa with the potential to sustain forward progressive motility, capacitate and subsequently participate in the cellular interactions required to achieve conception (1). Among the singular features that discriminate epididymal maturation from that of the preceding phases of gamete development (2) is that it is driven entirely by extrinsic factors in the absence of nuclear gene transcription and *de novo*, protein translation in the spermatozoa (3, 4). Indeed, it is widely held that the complex intraluminal microenvironment created by the epididymal epithelium serves as the key determinant in the functional transformation of spermatozoa (5, 6). Accordingly, the epididymal soma is characterized by a marked division of labor such that the proximal segments (initial segment, caput and corpus epididymis) promote sperm maturation, whereas the distal caudal segment supports sperm storage (1). Such functions are reflected in distinctive gene expression profiles (7–9) that, in turn, dictate segment-specific secretion of proteins and a range of additional biomolecules into the luminal fluid and thus establish the unique physiological compartments that affect sperm maturation and prolonged sperm survival (3, 10–12).

In recognition of the importance of epididymal function in governing sperm quality, this tissue has long been of interest as a potential site for contraceptive intervention (13–16). Conversely, the epididymis has also generated interest from the standpoint of therapeutic treatment strategies to combat sperm dysfunction associated with male factor infertility (17–20). The realization of both goals is predicated on resolution of the mechanistic basis by which the sperm proteome is so dramatically altered during the key developmental window of epididymal maturation. Among the potential mechanisms capable of mediating the bulk exchange of proteomic information to maturing spermatozoa, epididymosomes have emerged as attractive candidates (21–26). Epididymosomes represent a heterogeneous population of small membrane bound extracellular vesicles (EVs)^1^ (27–29) that are released from the epididymal epithelium via an apocrine secretory mechanism (30–32). This pathway is characterized by the formation of cytoplasmic protrusions along the apical margin of the principal epithelial cells (30). Following detachment, these “apical blebs” break down to release their contents, including epididymosomes, into the luminal environment (30) where they have the potential to interact with spermatozoa and mediate the transfer of a complex proteinaceous cargo to these cells (29).

The participation of epididymosomes in the alteration of the sperm proteome draws on a wealth of evidence that EVs, released from virtually all somatic tissues, can facilitate the delivery of a diverse macromolecular payload (comprising proteins, lipids, and nucleic acids) to recipient cells (33). It is also consistent with pioneering studies of Sullivan and colleagues who have demonstrated that bovine epididymosomes have the capacity to mediate the selective transfer of epididymal secretory proteins to homologous spermatozoa (26). At present however, the conservation of this form of intercellular communication for the *en masse*, delivery of proteins has yet to be substantiated in common laboratory models such as the rodents. To begin to address this challenge, we have surveyed the proteomic composition of epididymosomes isolated from different segments of the mouse epididymis using multiplexed tandem mass tag based relative quantification coupled with offline HPLC and LC-MS/MS. Further, we have exploited a co-culture system to demonstrate the uptake of biotinylated protein cargo from mouse epididymosomes primarily into the sperm head.

## EXPERIMENTAL PROCEDURES

### 

#### 

##### Reagents

Unless specified otherwise, all reagents were obtained from Sigma Aldrich (St. Louis, Mo) or Thermo Fisher Scientific (Waltham, MA) and were of research or mass spectrometry grade. Antibodies were purchased from the following suppliers: anti-DNM2 (PA5–19800; Thermo Fisher Scientific); anti-ADAM7, anti-BAG6, anti-clusterin, anti-HSPA2, anti-IZUMO1 (SC-25137, SC-365928, SC-166907, SC-79543, SC-79543; Santa Cruz Biotechnology, Dallas, TX); anti-ODF2, anti-PMSD7 (ab121023, ab11436; Abcam, Cambridge, UK); anti-GAPDH, anti-PDIA6 (G9545, HPA034653; Sigma-Aldrich); anti-PROM2 (NBP1–47941; Novus Biologicals, Littleton, CO). Anti-B4GALT1 and anti-MFGE8 antibodies were kindly provided by Professor Barry Shur, University of Colorado (Denver, CO).

##### Ethics Statement

All experimental procedures were conducted with the approval of the University of Newcastle's Animal Care and Ethics Committee (approval number A-2013–322), in accordance with relevant national and international guidelines. Inbred Swiss mice were housed under a controlled lighting regime (16L: 8D) at 21–22 °C and supplied with food and water *ad libitum*,. Prior to dissection, animals were euthanized via CO_2_ inhalation.

##### Mouse Epididymosome Isolation and Characterization

Mouse epididymosome isolation and validation of enrichment were conducted as previously described (34). Briefly, Swiss mice (adult males of at least 8 weeks of age) were euthanized and their vasculature immediately perfused with pre-warmed PBS to minimize the possibility of blood contamination. Epididymides were then removed, separated from fat and connective tissue and dissected into three anatomical regions corresponding to the caput, corpus and cauda. Luminal fluid was aspirated from each region by placing the tissue in a 500 μl droplet of modified Biggers, Whitten, and Whittingham media [BWW; pH 7.4, osmolarity 300 mOsm/kg (35, 36)] and making multiple incisions with a razor blade. The tissue was then subjected to mild agitation and the medium subsequently filtered through 70 μm membranes. This suspension was then sequentially centrifuged at increasing velocity (500 × *g*,, 5 min; 2000 × *g*,, 5 min; 4000 × *g*,, 5 min 8000 × *g*,, 5 min; 17,000 × *g*,, 20 min; and finally 17,000 × *g*, for an additional 10 min) to eliminate all cellular debris prior to the supernatant being layered onto a discontinuous iodixanol gradient (40%, 20%, 10%, and 5%; created by diluting 60% OptiPrep medium with a solution of 0.25 m sucrose, 10 mm Tris). The gradients were ultracentrifuged (100,000 × *g*,, 18 h, 4 °C), after which twelve equivalent fractions were collected, diluted in PBS and subjected to a final ultracentrifugation step (100,000 × *g*,, 3 h, 4 °C).

All isolated epididymosome fractions were characterized in accordance with the minimal experimental requirements for definition of extracellular vesicles (37), featuring analysis of their purity, particle size and overall homogeneity as previously described (34) (please see supplemental Fig. S1). Briefly, this included quantitative assessment of protein content and particle size heterogeneity of each of the twelve fractions; with the latter being achieved via measurement of mean particle size using dynamic light scattering (34). Additional immunoblot analyses were performed to determine the distribution of the exosome/epididymosome marker flotillin 1 (FLOT1) within each fraction, and a combination of FLOT1 and CD9 markers were also used to dual-label epididymosomes bound to aldehyde/sulfate latex beads (34). Finally, epididymosome preparations were also assessed via transmission electron microscopy to confirm the size and heterogeneity of the isolated populations. Notably, this experimental workflow was performed on all preparations of epididymosomes, irrespective of the downstream application. Moreover, as described below our proteomic analyses confirmed the presence of the top 50 proteins that are most commonly identified in exosomes, and whose identification is recommended as part of the minimal experimental requirements for definition of extracellular vesicles exosome protein markers (38).

To visualize changes in the epididymosome proteome, populations of epididymosomes from each epididymal segment (caput, corpus, cauda) were pooled from three animals to generate a single biological sample prior to labeling with cyanine dyes (with three such samples being analyzed in this study). Briefly, epididymosomes were lysed in rehydration buffer consisting of 7 m urea, 2 m thiourea, 4% CHAPS for 1 h on ice with regular vortexing. Extracted protein was quantified using a 2-D Quant kit in accordance with the manufacturer's instructions (GE Healthcare, Pittsburgh, PA) and a total of 75 μg of protein from each epididymosome sample was labeled with 600 pmol of appropriate cyanine-dye reagents (*i.e.*, either Cyanine3 or Cyanine5 NHS esters; Lumiprobe, Hunt Valley, MD) for 1 h on ice. Labeling reactions were quenched by addition of excess l-lysine (10 mm, 10 min on ice) after which differentially labeled epididymosome samples were combined (*i.e.*, either caput and corpus or corpus and cauda epididymosomes), prepared for resolution by 2D SDS-PAGE (39), and imaged using a Typhoon FLA 9500 laser scanner (GE Healthcare).

##### Epididymosome Protein Digestion and Labeling for Comparative and Quantitative Proteomic Analysis

Epididymosome preparations from each epididymal segment surveyed (caput, corpus, cauda) were pooled from twelve animals to generate a single biological replicate; with three such replicates being generated for analysis in this study. Epididymosome suspensions were then subjected to fractionation by dissolving in 200 μl of ice-cold 0.1 m Na_2_CO_3_ (pH 11.3) supplemented with protease and phosphatase inhibitors (Complete EDTA free; Roche, Basel, Switzerland) and probe tip sonicated at 4 °C for 2 × 20 s intervals prior to incubation for 1 h at 4 °C. Na_2_CO_3_ soluble proteins were isolated from insoluble-proteins by ultracentrifugation (100,000 × *g*, for 90 min at 4 °C) and dried (40). Both fractions were then dissolved in urea (6 m urea, 2 m thiourea) separately, reduced using 10 mm DTT (1 h, 56 °C, in the dark), and alkylated using 20 mm iodoacetamide (45 min, room temperature, in the dark). Proteins were digested using 1:50 ratio Lys-C/Trypsin to protein concentration, for 3 h at room temperature. The concentration of urea was then reduced below 0.75 m by adding 50 mm TEAB, pH 7.8 and incubated overnight at 37 °C. Peptides were desalted and cleaned up using a modified StageTip microcolumn and solid phase extraction (SPE) columns (Oasis PRIME HLB; Waters, Rydalmere, NSW, Australia), respectively (41). Quantitative fluorescent peptide quantification (Qubit protein assay kit; Thermo Fisher Scientific) was employed and 100 μg of each sample was labeled using tandem mass tags and comparative and quantitative analyses was performed in biological triplicate (42, 43) (TMT 10 plex labels; caput 1 = 127N, caput 2 = 127C, caput 3 = 128N, corpus 1 = 128C, corpus 2 = 129N, corpus 3 = 129C, cauda 1 = 130N, cauda 2 = 130C, cauda 3 = 131) (TMT-10plex 2 × kits; Thermo Fisher Scientific) (44). Digestion and tandem mass tag labeling efficiency was determined by LC-MS/MS (42). Samples were then mixed in 1:1 ratio and fractionated by hydrophilic interaction chromatography (HILIC; (45)) using a Dionex UltiMate 3000 capLC system (Dionex, Sunnyvale, CA) prior to nanoLC-MS/MS.

##### Tandem Mass Spectrometry (nanoLC-MS/MS) Comparative and Quantitative Analyses

NanoLC-MS/MS, was performed using a Dionex UltiMate 3000 nanoLC system (Dionex). HILIC fractionated peptides were suspended in buffer A (2% ACN/0.1% TFA) and directly loaded onto a 50 cm analytical column packed with Acclaim PepMap C18 2 μm sorbent. Peptides were eluted using a 110 min gradient from 7 to 40% buffer B (95% ACN, 0.1% TFA) at 250 nl min^−1^ and nanoelectrosprayed into a Q-Exactive Plus (Thermo Fisher Scientific). Precursor scan of intact peptides was measured in the Orbitrap by scanning from *m*,/*z*, 350–1500 (with a resolution of 70,000), the fifteen most intense multiply charged precursors were selected for HCD fragmentation with a normalized collision energy of 32.0, then measured in the Orbitrap at a resolution of 35,000. Automatic gain control targets were 3E6 ions for Orbitrap scans and 5E5 for MS/MS scans. Dynamic exclusion was employed for 15 s. Fragmentation data were converted to peak lists using Xcalibur version 4.027.19 (Thermo Fisher Scientific) and the HCD data were processed using Proteome Discoverer 2.1 (Thermo Fisher Scientific). MS spectra were then searched with Mascot V2.6 (accessed 05/06/2018) against all mouse entries in SwissProt (Release 2018 02; 16,976 entries). Mass tolerances in MS and MS/MS modes were 10 ppm and 0.02 Da, respectively; trypsin was designated as the digestion enzyme, and up to two missed cleavages were allowed. S-carbamidomethylation of cysteine residues was designated as a fixed modification. Variable modifications included were, oxidation of methionine, acetylation of lysine, deamidation of asparagine or glutamine and TMT labeling of amines and lysine. Interrogation of the corresponding reversed database was also performed to evaluate the false discovery rate (FDR) of peptide identification using Percolator based on q-values, which were estimated from the target-decoy search approach. To filter out target peptide spectrum matches (target-PSMs) over the decoy-PSMs, a fixed false discovery rate (FDR) of < 1% was set at the peptide level (46). Additional identification criteria consisted of a minimum of two uniquely matched peptides per protein and a Mascot score of ≥67 (47).

##### In Silico Analysis of Epididymosome Protein Cargo

*In silico*, analysis of epididymosome protein profiles was undertaken using a suite of techniques. Briefly, protein abundance data were assessed via volcano plots to visualize trends associated with differentially accumulating proteins in the epididymosomes sampled from opposing ends of the tract (*i.e.*, caput *versus*, cauda epididymal segments). Epididymosome protein datasets were also interrogated for enrichment of functional pathways using bioinformatic enrichment tools available via the Database for Annotation, Visualization and Integrated Discovery (DAVID; v6.8) (48, 49). Data sets were further curated based on sub-fertility phenotype terms using the MGI, Jackson Laboratory US, Genes and Genome Features database.

##### Validation of Quantitative Protein Accumulation in Epididymosomes

Orthogonal validation of the quantitative protein profiles generated by nanoLC-MS/MS was conducted using standard immunoblotting techniques. Representative proteins selected for analysis included those that exhibited highest expression in the caput or remained at relatively constant levels in epididymosomes sampled throughout the epididymis. All immunoblotting analyses were performed in biological triplicate, with each biological sample comprising epididymosome proteins pooled from a total of twelve mice. However, because of limitations in generating the volume of epididymosome material required for nano-LC-MS/MS, the protein used for immunoblot analyses was generated from different animals to those used for MS sequencing. Prior to analysis, pooled epididymosomes were solubilized by boiling in SDS extraction buffer (0.375 m Tris pH 6.8, 2% w/v SDS, 10% w/v sucrose, protease inhibitor mixture) at 100 °C for 5 min. Insoluble material was removed by centrifugation (17,000 × *g*,, 10 min, 4 °C) and soluble protein remaining in the supernatant was quantified using a BCA protein assay kit (Thermo Fisher Scientific). Equivalent amounts of protein (5 μg) were boiled in SDS-PAGE sample buffer (2% v/v mercaptoethanol, 2% w/v SDS, and 10% w/v sucrose in 0.375 m Tris, pH 6.8, with bromphenol blue) at 100 °C for 5 min, prior to be resolved by SDS-PAGE (150 V, 1 h) and transferred to nitrocellulose membranes (350 mA, 1 h). Membranes were then blocked and incubated with appropriate antibodies raised against target proteins. Briefly, blots were washed 3 times × 10 min with Tris-buffered saline with 0.1% (v/v) Tween-20 (TBST), before being probed with appropriate HRP-conjugated secondary antibodies. After three additional further washes, labeled proteins were detected using an enhanced chemiluminescence kit (GE Healthcare).

##### Transfer of Epididymosome Protein Cargo to Spermatozoa

Caput epididymal spermatozoa were isolated as previously described (50) in preparation for co-incubation with purified epididymosomes using methodology optimized for the *in vitro*, transfer of proteins between bovine epididymosomes and spermatozoa (51). Prior to co-culture, freshly isolated epididymosomes obtained from either the caput or cauda epididymal segments of 3 mice were pooled and resuspended in PBS to generate a single biological replicate. Epididymosome proteins were then labeled with a membrane impermeant, long-chain NHS-ester activated biotinylation reagent (EZ-Link sulfo-NHS-LC-Biotin, Thermo Fisher Scientific) (26.9 μm) for 30 min at room temperature followed immediately by overnight incubation at 4 °C. Following biotinylation, epididymosome suspensions were diluted into PBS supplemented with 50 mm glycine to arrest the biotinylation reaction (52) and subjected to ultracentrifugation (100,000 × *g*,, 3 h, 4 °C). The resultant epididymosome pellets were resuspended in modified BWW (pH 6.5) (34) in preparation for co-incubation with spermatozoa for 1 h at 37 °C in 5% CO_2_ with gentle agitation. These experiments were titrated such that the caput spermatozoa (∼10 × 10^6^ cells/ml) from one mouse were incubated with the equivalent of a single animal's epididymosomes (*i.e.*, the pooled epididymosome preparation described above was split into 3 equal portions prior to incubation with sperm). Following incubation, spermatozoa were washed three times by centrifugation (400 × *g*,, 3 min) in BWW to remove any unbound or loosely adherent epididymosomes, before a subset were set aside for affinity labeling with FITC-conjugated streptavidin to determine the localization of transferred proteins. The remaining cells were processed for total protein extraction to confirm the uptake of biotinylated proteins. Additional controls for this experiment included incubation of spermatozoa with FITC-conjugated streptavidin in the absence of prior exposure to epididymosomes, and direct biotinylation/FITC-streptavidin labeling of populations of caput spermatozoa (1 × 10^6^) for 1 h at 37 °C in 5% CO_2_. These experiments were performed in triplicate, with independent biological samples (*i.e.*, spermatozoa and epididymosomes) having each been isolated from different animals and a minimum of 100 spermatozoa were assessed/replicate within each treatment group.

To assess the putative involvement of candidate epididymosome ligands (*i.e.*, milk fat globule-EGF factor 8; MFGE8) in epididymosome-sperm interaction, the experimental procedure described above was replicated using biotinylated caput epididymosomes pre-incubated with anti-MFGE8 antibodies (10 μg/ml) for 1 h at room temperature with constant slow rotation. After incubation, the epididymosomes were washed free of unbound antibody with PBS, collected by ultracentrifugation (100,000 × *g*,, 1.5 h, 4 °C) and subsequently assessed for the efficiency with which they were able to transfer biotinylated proteins to the postacrosomal sheath of caput spermatozoa, using equivalent techniques to those described above. Controls for this experiments included the omission of the anti-MFGE8 antibody as well as its substitution with an irrelevant antibody control (*i.e.*, anti-GAPDH antibodies).

##### Immunofluorescence and Electron Microscopy

Immunolocalization of MFGE8 was performed on isolated spermatozoa and epididymal tissue sections in accordance with previously described protocols (53, 54). Briefly, spermatozoa were settled onto poly-l-lysine-coated coverslips overnight at 4 °C. All subsequent incubations were performed at room temperature in a humidified chamber, and all antibody dilutions and washes were conducted in PBS containing 0.1% Tween-20 (PBST). Fixed cells were permeabilized in 0.2% Triton X-100/PBS for 10 min and blocked in 3% (w/v) BSA in PBST for 1 h. Coverslips were then sequentially labeled with anti-MFGE8 antibodies (diluted 1:100) for 1 h at room temperature. After incubation, coverslips were washed three times, and then incubated in goat anti-rabbit Alexa Fluor 488 (diluted 1:200) secondary antibody for 1 h at room temperature. Cells were then washed and counterstained in 4′,6-diamidino-2-phenylindole (DAPI) before mounting in antifade reagent (Mowiol 4–88). Labeled cells were viewed on an Axio Imager A1 microscope (Carl Zeiss MicroImaging, Oberkochen, Germany) equipped with epifluorescent optics and images captured with an Olympus DP70 microscope camera (Olympus, Shinjuku, Tokyo, Japan). Alternatively, mouse epididymal tissue was fixed in Bouin's fixative, embedded in paraffin wax and sectioned at 5 μm thickness. Sections were de-paraffinized, rehydrated, and antigen retrieval was performed by boiling the slides for 10 min in 10 mm Tris (pH 10). Sections were blocked in 3% w/v BSA/PBST for 1 h at room temperature, after which they were incubated with anti-MFGE8 (diluted 1:200 with 1% w/v BSA/PBST) overnight at 4 °C. Sections were then washed and incubated with fluorescent-conjugated secondary antibody, Alexa Fluor 594 goat anti-rabbit IgG (1:200 with 1% w/v BSA/PBST), for 1 h at room temperature. DAPI counterstaining was conducted for 3 min. Sections were then mounted in Mowiol and viewed on an Axio Imager A1 microscope (Carl Zeiss) as describe above.

To visualize *in situ*, epididymosome-sperm interactions, mouse caput epididymal tissue was fixed in 4% (w/v) paraformaldehyde containing 0.5% (v/v) glutaraldehyde as previously described (54). The tissue was then processed via dehydration, infiltration and embedding in LR White resin. Sections (100 nm) were cut with diamond knife (Diatome Ltd., Bienne, Switzerland) on an EM UC6 ultramicrotome (Leica Microsystems, Vienna, Austria) and placed on 150-mesh nickel grids. For MFGE8 detection, epididymal sections were blocked in 3% (w/v) BSA in PBS at 37 °C for 30 min before being subjected to overnight incubation with anti-MFGE8 antibody (MBS2004903, diluted 1:20 in 1% BSA in PBS) at 4 °C. After incubation, sections were washed free of primary antibody via immersion in five sequential changes of 1% BSA in PBS (5 min each), and then incubated with appropriate secondary antibody conjugated to 10 nm gold particles (G7402, diluted 1:10 in 1% BSA in PBS) for 2 h at 37 °C. After washing, labeled sections were counterstained in 2% (w/v) uranyl acetate in 40% (v/v) methanol for 20 min. Micrographs were captured on a JEOL-100CX transmission electron microscope (JEOL, Tokyo, Japan) at 80kV. These experiments were performed in triplicate, with independent biological samples having each been isolated from different animals.

##### Experimental Design and Statistical Rationale

For all experiments, individual biological replicates comprised pooled preparations of epididymosomes isolated from the appropriate epididymal segment (caput, corpus, cauda) of between three - twelve animals. Pooling of material from this number of animals was necessitated based on recovering enough protein for each downstream application. Three such biological replicates were used for tandem mass tag labeling to generate our primary epididymosome proteomic inventory and to facilitate comparative and quantitative proteomic analyses. Epididymosome proteins were identified as being differentially accumulated between epididymal segments if they experienced a fold change of ≤ −1.5 or ≥ 1.5; *p*, < 0.05. Immunoblotting of candidate epididymosome proteins was performed (*n*, = 3) in order validate our quantitative epididymosome protein abundance data. Similarly, additional replicates were also employed for confirmation of epididymosome-mediated transfer of biotinylated proteins to mouse spermatozoa (*n*, = 3). Please see supplemental Fig. S2 for full details of experimental design including the number of animals/replicates used per experiment.

## RESULTS

### 

#### 

##### Global Proteomic Analysis of Mouse Epididymosomes

Mouse epididymosomes were recovered separately from the caput, corpus and cauda epididymides prior to being subjected to Lys-C/Trypsin digestion, TMT labeling and MS analysis. Using stringent identification criteria (described above), this experimental strategy identified a complex proteomic cargo comprising a total of 1640 unique proteins in epididymosomes sampled from across all three epididymal segments. Among these proteins, an average number of 11.8 unique peptide matches were generated per protein; representing an average peptide coverage of ∼30% per protein (Table I, supplemental Table S1).

**Table I:** Summary of mouse epididymosome proteome data set

	Total proteins identified	Av. peptide hits/protein	Av. unique peptide hits/protein	Av. protein coverage (%)	Number of differentially accumulated proteins (fold change + 1.5)		
					Corpus vs Caput	Cauda vs Corpus	Cauda vs Caput
Mouse epididymosomes	1640	13.1	11.8	29.9	146	344	474

**Fig. 1.**
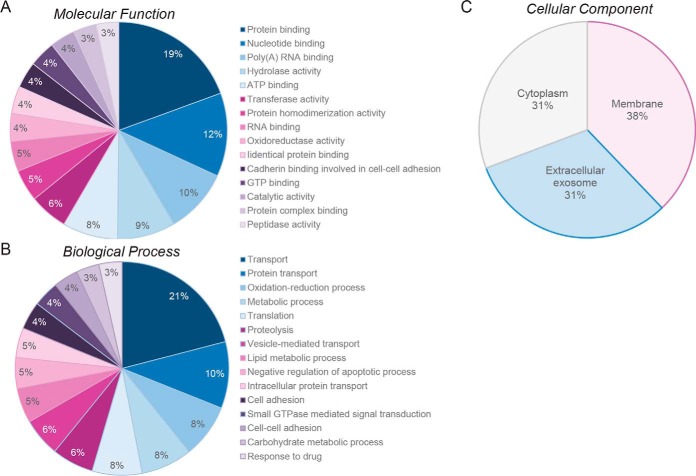
**GO annotation of mouse epididymosome protein cargo.** Among the core inventory of 1640 epididymosome-associated proteins identified in this study, a total of 1564 (95%) were able to be annotated according to GO information based on (*A*,) molecular function, (*B*,) biological process, and (*C*,) cellular component. The percentage of epididymosome proteins mapping to the 15 highest ranked (based on number of assigned proteins) (*A*,) molecular function and (*B*,) biological process categories are depicted. (*B*,) Similarly, the percentage of epididymosome proteins mapping to the 3 highest ranked cellular component categories are also shown.

Provisional interrogation of this global epididymosome proteome on the basis of shared functional classification using DAVID Gene Ontology (GO) annotation tools (version 6.8) returned dominant terms of “protein binding” (GO identifiers: 5515, 32403, 42802, 42803, 98641), “nucleotide binding” (GO identifier: 166, 3723, 5524, 5525, 44822), “oxidoreductase activity” (GO identifier: 16491) and “catalytic activity” (GO identifiers: 3824, 16,491, 16787) among the top 15 GO molecular function categories when ranked on the basis of number of annotated proteins (Fig. 1*A*,, supplemental Table S2). Similarly, in terms of GO biological process categories, notable enrichment was identified in the broad term of “transport” (GO identifier: 6810), as well as the more specific terms of “protein transport” (GO identifier: 15031), “vesicle-mediated transport” (GO identifier: 16192) and “intracellular protein transport” (GO identifier: 6886) (Fig. 1*B*,, supplemental Table S2). Other notable GO biological process categories of direct relevance to epididymal physiology/function included: “oxidation-reduction process” (GO identifiers: 6979, 55114), “proteolysis” (GO identifiers: 6508), “metabolic process” (GO identifiers: 5975, 6629, 6631, 8152), “binding of sperm to zona pellucida” (GO identifier: 7339), and cell adhesion (GO identifiers: 7155, 98609) (supplemental Table S2). As might be expected of epididymosome-encapsulated cargo, the dominant GO cellular component categories were identified as “membrane,” “extracellular exosome,” and “cytoplasm” with 983, 815, and 799 proteins mapping to these respective categories (Fig. 1*C*,, supplemental Table S2).

Additional curation of the epididymosome proteome on the basis male sub-fertility phenotypes identified at least 98 proteins whose dysregulated expression has previously been linked with male fertility phenotypes and/or defective sperm production/function (supplemental Table S3). Aside from the broad categories of “male infertility” and “reduced male fertility,” common pathologies attributed to the loss/dysregulation of male fertility in this sub-class of proteins included: “reproductive system phenotype,” “abnormal epididymis morphology,” “abnormal male reproductive system physiology,” and “abnormal sperm morphology/physiology.” Presumably reflecting conserved protein expression in both the testes and epididymis, “abnormal testicular morphology,” “decreased testis weight,” and associated defects in “spermatogenesis” and “germ cell number” also featured prominently among the defined pathological lesions associated with these proteins (supplemental Table S3).

##### Conservation of Epididymosome Cargo

**Fig. 2.**
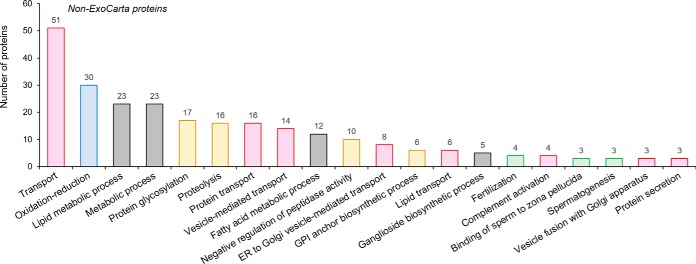
**GO biological processes associated with mouse epididymosome proteins not represented in the ExoCarta database.** A total of 288 proteins were identified in mouse epididymosomes that did not correspond with entries curated in the ExoCarta database. These proteins were annotated according to GO information based on biological process and the 20 highest ranked (based on number of assigned proteins) categories are depicted. Colored columns represent those proteins clustered to the GO terms related to transport (pink), oxidation-reduction (blue), metabolism (gray), protein synthesis/degradation (yellow), and sperm function (green).

In view of the potential for overlapping distribution of proteins between the testicular and extra-testicular regions of the male reproductive tract, we sought to confirm that the epididymosome proteins identified herein do indeed constitute those expected of an enriched exosome population. For this purpose, our complete inventory of identified proteins were used to interrogate ExoCarta, a web-based database featuring a comprehensive assemblage of exosomal cargo identified across multiple tissues and organisms (38). In contrast to our GO analysis in which a conservative 50% (815/1640) of the identified proteins mapped to the cellular component category of “extracellular exosome,” (Fig. 1*B*,, supplemental Table S2), our survey of the ExoCarta database identified as many as 1352/1640 proteins (representing 82%) that have previously been identified among exosome-borne cargo (supplemental Table S4). Notably, this conserved list featured many proteins, such as those implicated in transcription and translation, which one may not normally consider to be secreted to the extracellular environment (supplemental Table S4). It also comprised all of the top 50 exosome protein markers (38), as well as 92 out of the top 100 proteins that are most commonly identified in exosomes, and whose identification is recommended as part of the minimal experimental requirements for definition of extracellular vesicles (37). Examples included: tetraspanins (CD9, CD63, CD81, CD151), integrins (ITGA1, ITGA2, ITGA3, ITGA5, ITGAM, ITGAV, ITGB1, ITGB2), endosome/membrane binding proteins (TSG101, ANXA2, ANXA5, 22 × RAB family members), signal transduction/scaffolding proteins (syntenin-1, syntenin-2), and molecular chaperones (HSPA8, HSP90AA1) (supplemental Table S4). In contrast, functional annotation of those epididymosome proteins that did not correspond to entries in the ExoCarta database revealed enrichment in the GO biological process categories that one might expect to be restricted to the male reproductive tract, including “spermatogenesis,” “binding of sperm to zona pellucida,” and “fertilization” (GO identifiers: 7283, 7339, 7338) (Fig. 2, green columns). In addition to an abundance of proteins linked to transport, this latter subset of proteins also featured substantial enrichment of GO categories known to be associated with modification of the maturing sperm proteome, such as “protein glycosylation” (GO identifier: 6486), “proteolysis” (GO identifier: 6508), “peptidase activity” (GO identifier: 10466), and “GPI anchor” (GO identifier: 6506) (Fig. 2, yellow columns).

In seeking to explore the conservation of epididymosome proteomic cargo across species, we also surveyed published proteomic lists generated for epididymosomes isolated from the bovine (24), human (55), and ovine (56) epididymis. Although such information has yet to be curated in ExoCarta, the most comprehensive of these datasets (*i.e.*, that generated from the bovine model) comprises an impressive 762 proteins that map to epididymosomes isolated from the caput and/or caudal epididymal segments (24). Among these proteins, we were able to confirm the conservation of at least 367/762 (48%) in both mouse and bovine epididymosomes (supplemental Table S4). Although considerable overlap was evident across most functional protein categories, we recorded particularly high conservation among the subset of ribosomal and proteasomal protein cargo, as well as those proteins mapping to the broad functional categories of “transporters and protein trafficking,” “chaperone molecules,” and “enzymes.” We also noted enrichment of proteins associated with “defense” such as the beta-defensin family and many of the complement dependent (CD) proteins that have previously been characterized in bovine epididymosomes. In assessing the more modest proteomic profiles generated for human and ram epididymosomes we were able to identify relatively high levels of conservation. Indeed, our data comprised at least 101/146 (69%) and 25/28 (89%) of those proteins previously identified in human (55) and ram (56) epididymosomes, respectively (supplemental Table S4).

As an extension of this analysis, we also explored the similarity of the epididymosome proteome with that of an independently published mouse sperm proteome (57) (supplemental Table S4). Notwithstanding technical limitations imposed by incomplete sequence coverage of the sperm proteome and conversion of UniProt accession numbers to corresponding Gene Name IDs (limiting our comparison to 1560/1640 epididymosome proteins), this approach reveled the conservation of 589, 624, and 407 epididymosome proteins within the caput, corpus, and cauda sperm proteomes, respectively.

##### Relative Quantification of Differential Protein Accumulation into Epididymosomes

**Fig. 3.**
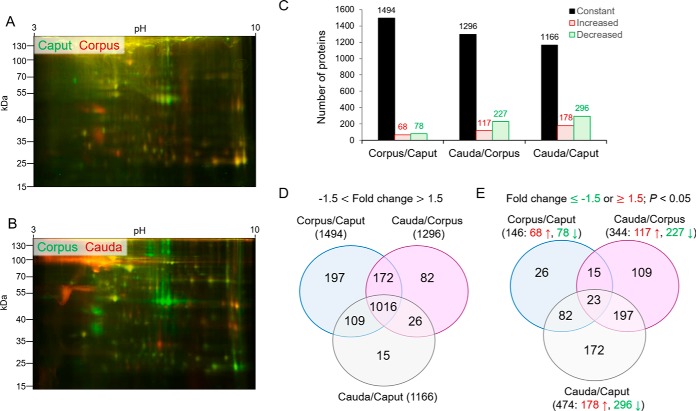
**Comparative analysis of mouse epididymosome protein abundance.** The differential accumulation of epididymosome protein cargo was assessed via cyanine-dye labeling of epididymosome protein extracts, recovered from (*A*,) the proximal (caput *versus*, corpus) and (*B*,) distal (corpus *versus*, cauda) epididymal segments. Labeled proteins extracted from the two different epididymosome populations were mixed, prepared for resolution by 2D SDS-PAGE and visualized via the use of a Typhoon FLA 9500 laser scanner. This experiment was replicated three times using paired epididymosome samples (*i.e.*, either caput and corpus or corpus and cauda epididymosomes) and depicted are representative merged gel images for (*A*,) caput (cyanine3-labeled, pseudo-colored in green) and corpus (cyanine5-labled, red) epididymosome proteins or alternatively, (*B*,) corpus (cyanine3-labeled, pseudo-colored in green) and cauda (cyanine5-labled, red) epididymosome proteins. *C–E*,, The relative abundance of epididymosome proteins was also determined via assessment of TMT reporter ion intensity in samples isolated from the caput, corpus and cauda segments of the mouse epididymis. For this analysis a threshold of ± 1.5 fold change (*p*, < 0.05) was set as the basis for assignment of differentially accumulated proteins. *C*,, The overall number of proteins that experienced no-change (black columns), increased (red columns), or decreased (green columns) accumulation in each epididymosomes population are shown. Similarly, the conservation of proteins that were either (*D*,) unchanged or (*E*,) experienced ≥ 1.5-fold increase (red font, ↑) or decrease (green font, ↓) change among different epididymosome sub-populations are also shown.

Having established the overall proteomic composition of mouse epididymosomes, we next investigated changes encountered in different epididymal segments. These studies were initiated via labeling of epididymosome proteins with Cy-dyes prior to their resolution by 2D SDS-PAGE to visually compare their proteomic profiles. Using this approach, we documented a relatively high degree of commonality in the proteomic cargo of epididymosomes from the proximal epididymal segments (*i.e.*, caput *versus*, corpus epididymis) (Fig. 3*A*,). By contrast, more overt qualitative and quantitative differences were noted in the proteomic cargo of corpus *versus*, cauda epididymosomes (Fig. 3*B*,). Accordingly, this analysis was expanded to include an interrogation of the intensity of reporter ions tagged to each peptide to determine the differential accumulation of proteins into epididymosomes recovered from the caput, corpus and cauda segments of the mouse epididymis. In this analysis, an arbitrary threshold of ± 1.5-fold change (*p*, < 0.05) was selected as the basis for assignment of differentially accumulated proteins. Using this criterion, we identified a substantial number of proteins whose relative abundance remained consistent in all epididymosome sub-populations surveyed (Fig. 3*C*,). Indeed, in considering the cumulative changes in protein abundance between the proximal (caput) and distal (cauda) epididymosome sub-populations, ∼71% of the total proteome were detected at equivalent levels (Fig. 3*C*,, 3*D*,).

**Fig. 4.**
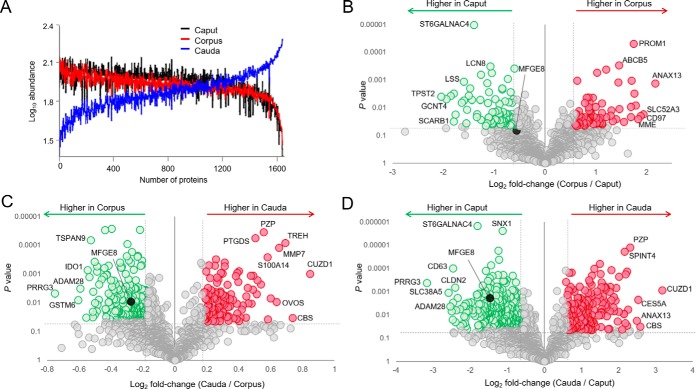
**Plots depicting fold changes associated with differentially accumulated epididymosome proteins.** *A*,, Protein abundance cluster analysis depicting patterns of relative abundance for each of the 1640 epididymosome proteins quantitated in this study. The *y*, axis represents the average reporter ion abundance (log_10_ scale) determined for each of the 1640 proteins identified in this study (*x*, axis). *B–D*,, Volcano plots were constructed to demonstrate the log_2_ fold change (*x*, axis) and probability score (*y*, axis) of proteins that were determined to be differentially accumulated in epididymosomes isolated from the (*B*,) corpus *versus*, caput, (*C*,) cauda *versus*, corpus, and (*D*,) cauda *versus*, caput epididymal segments. Thresholds of ± 1.5-fold change (*p*, < 0.05) in TMT reporter ion intensity were implemented to identify proteins subject to differential accumulation in epididymosomes from different epididymal segments. Several epididymosome proteins that experienced prominent fold-changes between epididymal segments are annotated as is the MFGE8 protein that was targeted for additional characterization.

Notwithstanding the conservation of this subset of core epididymosome proteins, we did document apparent gradients of accumulation/reduction associated with the abundance of many of the epididymosome proteins. As anticipated, these changes were more pronounced when considered across the entire epididymal tubule (*i.e.*, caput *versus*, cauda). Indeed, we identified 474 (∼29%) proteins that were differentially accumulated in cauda epididymosomes *versus*, those sampled from the caput; including, 296 proteins that were under-represented, and a further 178 proteins that were over-represented in the cauda samples (Fig. 3*E*,). In evaluating the temporal appearance of these changes in the epididymosome proteome, the majority coincided with the transition from the corpus to the cauda epididymal segments as opposed to between the more proximal caput to corpus segments. Such findings accord with our Cy-dye labeling data (Fig. 3*A*,, 3*B*,) as well as the physiological roles of the different epididymal segments; with the caput and corpus participating in sperm maturation and the cauda fulfilling a key role in sperm storage/maintenance (58). In extending support for these divergent roles, abundance cluster analyses confirmed that epididymosomes from the caput and corpus possess a strikingly similar protein abundance profile (Fig. 4*A*,). Conversely, the population of cauda epididymosomes were characterized by an almost reciprocal protein abundance profile (Fig. 4*A*,).

These quantitative changes included several proteins that experienced ≥5-fold changes between the sub-populations of caput and cauda epididymosomes (Fig. 4*B*,–4*D*, and supplemental Table S1). One of the most dominant among these was CUB and zona pellucida-like domain-containing protein (CUZD1), a protein that was detected at levels of ∼9-fold higher in the cauda segment *versus*, that of the caput, respectively (Fig. 4*D*, and supplemental Table S1). Conversely, proteins such as PRRG3, SCL38A5, B4GALT4, ADAM28, ADAM7, RNASE10 were characterized by an apparent reduction of between 5- and 9-fold in epididymosome fractions over the same epididymal segments (Fig. 4*D*, and supplemental Table S1).

##### Gene Ontology Analysis of Differentially Accumulated Epididymosome Proteins

**Fig. 5.**
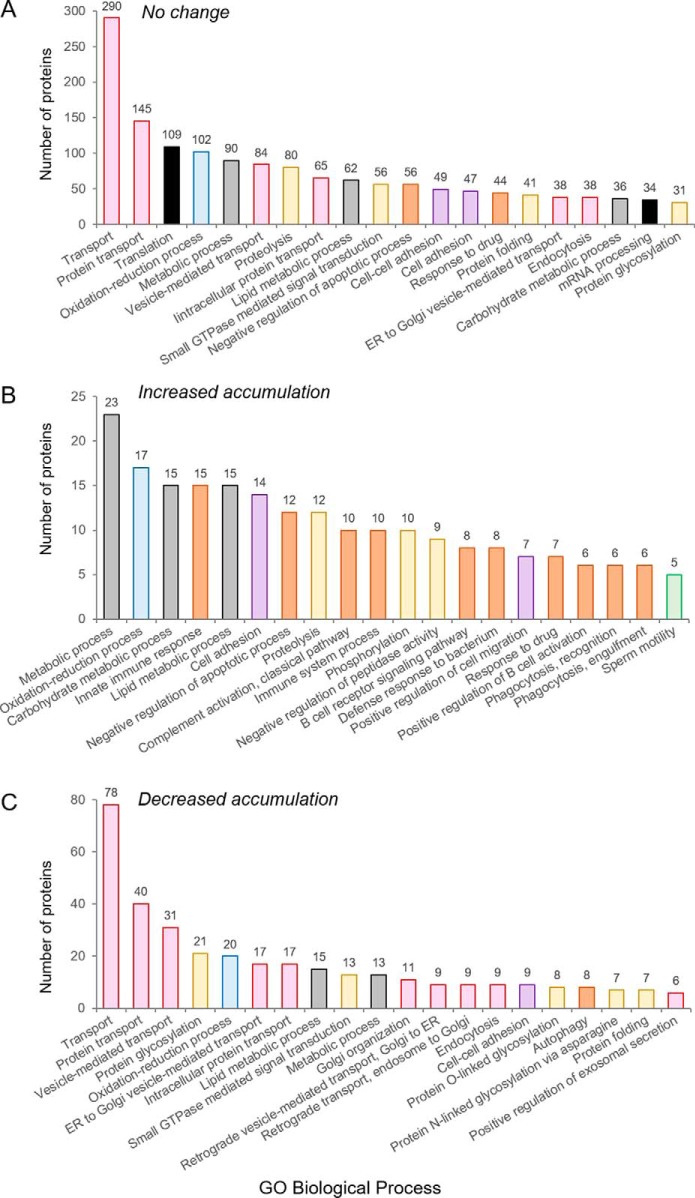
**GO annotation of differentially accumulated epididymosome protein cargo.** Epididymosome proteins were segregated based on relative levels of abundance in cauda *versus*, caput epididymosomes into those that experienced (*A*,) no change, (*B*,) increased accumulation, or (*C*,) decreased accumulation. Proteins within each category were subsequently annotated according to GO biological process. Shown are the top 20 biological processes assigned based on the number of mapped proteins. Colored columns represent those proteins clustered to the GO terms related to transport (pink), metabolism (gray), oxidation-reduction (blue), protein interactions/catabolism (yellow), cell-cell adhesion (purple) and cellular responses to stress, etc (orange). Additional, dominant GO categories are denoted by black columns.

To investigate the functional characteristics of conserved proteins as opposed to those that were differentially accumulated into populations of caput and cauda epididymosomes, each subset was classified according to their known, or predicted, biological processes using Gene Ontology categories (48, 49) (Fig. 5). As previously documented following curation of the entire epididymosome proteome (Fig. 1), many of the proteins that were detected at equivalent levels in caput and cauda epididymosomes mapped to the dominant GO biological process categories of “transport” (GO identifier: 6810), “protein transport” (GO identifier: 15031), “vesicle-mediated transport” (GO identifier: 16192), “intracellular protein transport” (GO identifier: 0006886), “ER to Golgi vesicle-mediated transport” (GO identifier: 6888), and “endocytosis” (GO identifier: 6897) (Fig. 5*A*,; pink columns). Notably, these transport categories also dominated the GO profile of proteins that experienced a decreased abundance in cauda *versus*, caput epididymosomes (Fig. 5*C*,; pink columns). In contrast, those epididymosome proteins for which a positive gradient of accumulation was documented between the caput and caudal segments, mapped to common GO biological process categories of protein degradation/modification: “proteolysis,” “phosphorylation,” “negative regulation of peptidase activity” (GO identifiers: 6508, 16310, 10466) (Fig. 5*B*,; yellow columns); metabolism: “metabolic process,” “carbohydrate metabolic process,” and “lipid metabolic process” (GO identifiers: 8152, 5975, 6629) (Fig. 5*B*,, gray columns); “oxidation-reduction process” (GO identifier: 55114) (Fig. 5*B*,; blue columns); as well as proteins that clustered into GO biological processes synonymous with immunological responses: “innate immune response,” “complement activation,” “immune system process,” “B-cell receptor signaling pathway,” and “defense response to bacterium,” “positive regulation of B cell activation,” “phagocytosis, recognition/engulfment” (GO identifiers: 45087, 6958, 2376, 50853, 42742, 50871, 6910, 6911) (Fig. 5*B*,; orange columns). In the opposing subset of proteins characterized by lower abundance in cauda epididymosomes, the prevailing GO biological processes were clearly differentiated, featuring numerous categories associated with vesicle transport/secretion, which in addition to those described above, included “retrograde vesicle-mediated transport, Golgi to ER/endosome to Golgi” and “positive regulation of exosomal secretion” (GO identifiers: 6890, 42147, 1903543) (Fig. 5*C*,; pink columns).

##### Validation of Differentially Accumulated Epididymosome Proteins

**Fig. 6.**
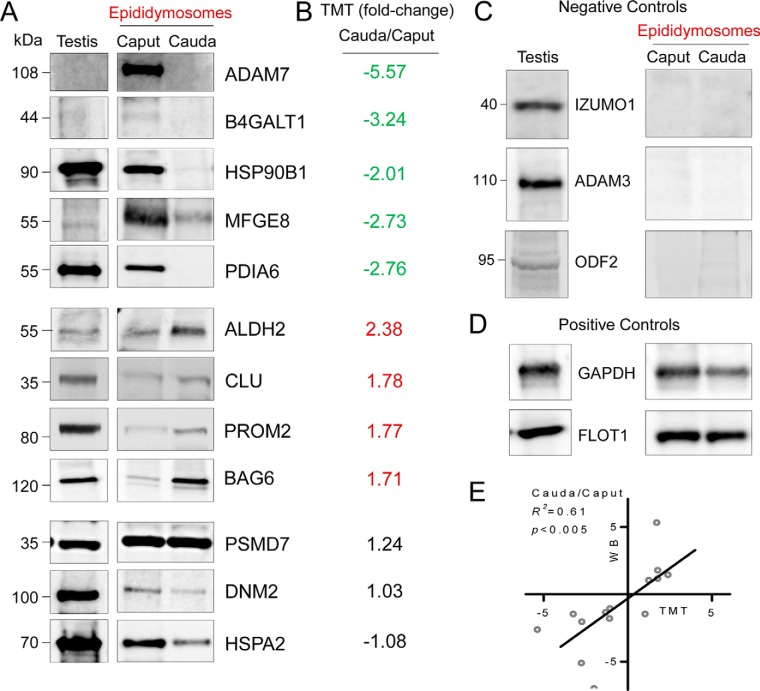
**Immunoblot validation of the abundance of differentially accumulating epididymosome proteins.** *A*,, Quantitative MS data were validated via immunoblotting of differentially accumulating proteins. Candidate proteins included representatives with the highest abundance (according to TMT reporter ion intensity) in epididymosomes from the proximal segment of the epididymis (caput) (ADAM7, B4GALT1, HSP90B1, MFGE8, PDIA6) in addition to proteins exhibiting increasing accumulation in cauda epididymosomes (*i.e.*, ALDH2, CLU, PROM2, BAG6), and those that remained at relatively constant levels in epididymosomes sampled throughout the epididymis (PSMD7, DNM2, HSPA2). *B*,, Corresponding MS quantification data are presented. *C*,, Negative controls included sperm proteins of testicular origin (IZUMO1, ADAM3, ODF2), *D*,, whereas positive controls included validated epididymosome/exosome proteins (GAPDH, FLOT1). Analyses were performed in triplicate using biological samples comprising pooled epididymosomes purified from 12 mice and representative immunoblots are depicted. *E*,, A linear regression was performed to compare the quantification data obtained via TMT (*x*, axis) and immunoblotting (*y*, axis) analyses for each of the targeted epididymosome proteins, revealing significant correlation (*R*,^2^ = 0.61; *p*, < 0.005) between these data sets.

To confirm the differential accumulation of proteins into epididymosomes, 12 candidate proteins were selected for orthogonal targeted validation via immunoblotting. Most of these proteins were selected based on highest abundance in the caput epididymosomes before experiencing reduced abundance in the cauda segment of the epididymis (*i.e.*, ADAM7, B4GALT1, HSP90B1, MFGE8, PDIA6). However, this analysis also included proteins exhibiting the reciprocal trend of increasing accumulation in cauda epididymosomes (*i.e.*, ALDH2, CLU, PROM2, BAG6), as well as those that remained at relatively constant levels in epididymosomes sampled throughout the epididymis (PSMD7, DNM2, HSPA2). All immunoblotting experiments were performed in triplicate using pooled biological samples (*n*, = 3 animals/sample) differing from those employed for MS analyses and, in each experiment, flotillin 1 (FLOT1) was employed as an endogenous control to normalize the abundance levels of targeted proteins (Fig. 6*D*,). This analysis confirmed the differential accumulation of 9 of the targeted epididymosome proteins, as well as the equivalent abundance of 2 of the remaining candidates (Fig. 6*A*,); with each of these proteins characterized by an accumulation profile that closely paralleled the trends identified by MS analyses (Fig. 6*B*,). The one exception was that of HSPA2, which was under-represented in the cauda epididymosomes via immunoblotting, yet recorded at equivalent levels in caput and cauda epididymosomes via MS analysis (Fig. 6*A*,, 6*B*,). Moreover, we were unable to detect the presence of selected sperm proteins of testicular origin that were included as negative controls (*i.e.*, IZUMO1, ADAM3, ODF2) (Fig. 6*C*,). Accordingly, a linear regression comparing the fold-changes recorded for each of these targets revealed significant correlation (*R*,^2^ = 0.61; *p*, < 0.005) between the quantification data obtained via TMT and immunoblotting analyses (Fig. 6*E*,). Together, such findings support the accuracy of our data in reflecting the spatial patterns of mouse epididymosome proteomic signatures.

##### Accumulation of Biotinylated Epididymosome Cargo into Spermatozoa

**Fig. 7.**
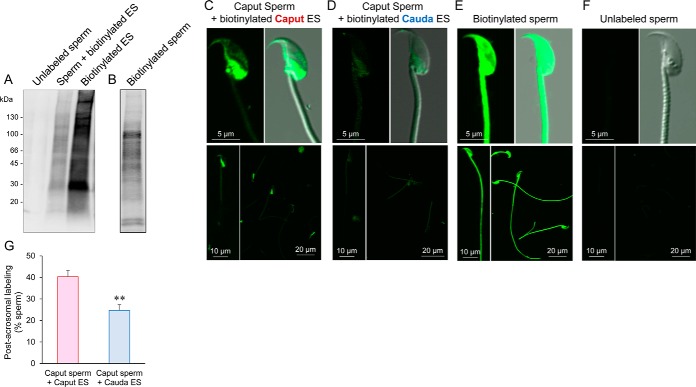
**Examination of the transfer of biotinylated proteins to spermatozoa after co-incubation with epididymosomes.** The ability of mouse epididymosomes to transfer protein cargo to spermatozoa was assessed via labeling of caput epididymosomes with a membrane-impermeant biotin reagent. The biotinylated epididymosomes (biotinylated ES) were co-incubated for 1 h with spermatozoa isolated from the caput epididymis, after which the cells were washed thoroughly and split into two fractions in preparation for assessment of biotinylated protein uptake via either (*A*,, *B*,) immunoblotting or (*C*,) affinity labeling with HRP- or FITC-conjugated streptavidin, respectively. *D*,, To explore the specificity of epididymosome-sperm interactions, an equivalent experiment was performed in which caput spermatozoa were incubated with epididymosomes isolated from the cauda epididymis. *E*,, *F*,, Controls included equivalent populations of spermatozoa incubated under identical conditions in the absence of epididymosomes. These cells were either left in an unlabeled state (*A*,, *F*,: Unlabeled sperm) to confirm the absence of auto-fluorescence or incubated directly with biotin reagent (*B*,, *E*,) to confirm the specificity of epididymosome-mediated protein transfer. (*G*,) Additionally, the efficacy of biotinylated protein transfer into the post-acrosomal sheath of the sperm head was assessed following co-incubation of caput spermatozoa with either caput (pink column) or cauda (blue column) epididymosomes. These analyses were performed in triplicate using biological samples comprising pooled epididymosomes purified from three mice. During co-incubation, spermatozoa and epididymosomes were titrated to a ratio of 1:1; that is, aliquots of spermatozoa recovered from one animal (∼10 × 10^6^ cells) were inseminated with epididymosomes also equating to those isolated from a single animal. A minimum of 100 spermatozoa were assessed/replicate within each treatment group and graphical data are presented as means ± S.E.; ** *p*, < 0.01. Representative immunoblots and immunofluorescence images are shown.

Having confirmed substantial changes in the overall profile and relative levels of proteins present within mouse epididymosomes, we next sought to determine whether these vesicles were capable of delivering this macromolecular cargo to spermatozoa. Specifically, we applied an optimized co-incubation strategy (34) to track the transfer of biotinylated epididymosome protein cargo into mouse spermatozoa sampled from the caput epididymis. This analysis revealed significant accumulation of biotinylated protein into the sperm proteome (Fig. 7*A*,). Of note, the epididymosome-mediated transfer of biotinylated protein appeared to be selective such that at 1 h postincubation with caput epididymosomes, these cargo were predominantly localized within the post-acrosomal sheath of the head of ∼40% of the sperm population (Fig. 7*C*,). Additional labeling, albeit far less intense, was detected within the anterior acrosomal region of the head of these spermatozoa (Fig. 7*C*,). Alternatively, a small number of cells (*i.e.*, < 15%) were characterized by punctate labeling that was either distributed throughout the sperm head or restricted to the sub-acrosomal ring; however, we rarely (*i.e.*, < 5%) observed any labeling of the sperm flagellum. To extend our analysis of the specificity of epididymosome-sperm interaction, we performed a heterologous co-incubation assay in which caput sperm were incubated with epididymosomes recovered from the cauda epididymis. Thereafter, we recorded the transfer of biotinylated protein cargo primarily into the post-acrosomal sheath of the sperm head, an equivalent domain to that witnessed following incubation with caput epididymosomes (Fig. 7*D*,). Notably however, both the intensity of the biotin labeling (Fig. 7*D*,), and the number of spermatozoa incorporating this label were significantly reduced compared with caput sperm incubated with caput epididymosomes (*i.e.*, 24.7 ± 2.7 *versus*, 40.3 ± 2.9%, respectively; *p*, < 0.01) (Fig. 7*G*,). To control for the possibility of nonspecific labeling because of the presence of unreacted biotin reagent, spermatozoa were also subjected to direct biotinylation, revealing a distinct pattern of labeling that was uniformly distributed across all sperm domains (Fig. 7*E*,). Similarly, the profile of sperm proteins targeted for direct biotinylation also differed from that present in either biotinylated epididymosomes or in sperm lysates following their co-incubation with biotinylated epididymosomes (Fig. 7*B*,). Alternatively, we failed to detect any endogenous biotin labeling within naive populations of sperm incubated in the absence of epididymosomes (Fig. 7*A*,, 7*F*,).

**Fig. 8.**
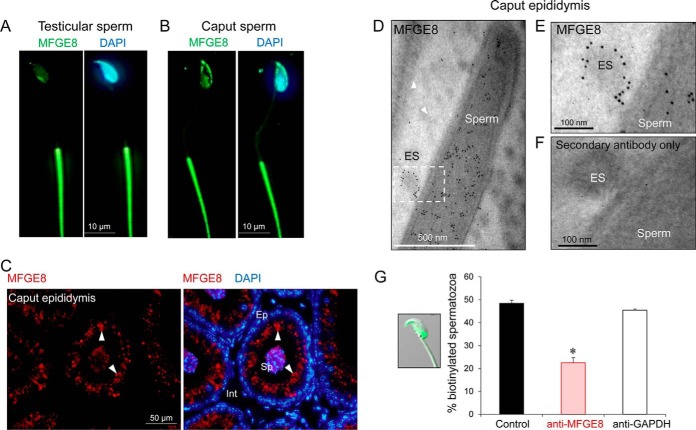
**Characterization of the exosome marker, MFGE8, in mouse epididymosomes.** Anti-MFGE8 antibodies were used to localize the protein in populations of (*A*,) testicular spermatozoa and those isolated from the (*B*,) caput epididymis. Spermatozoa were sequentially labeled with an appropriate Alexa Fluor 488-conjugated secondary antibody (green) and counterstained with the nuclear stain DAPI (blue). *C*,, Similarly, anti-MFGE8 antibodies were also used to label caput epididymal tissue sections, prior to the application of an Alexa Fluor 594-conjugated secondary antibody (red) and DAPI counterstain (blue). Arrowheads indicate supranuclear labeling of epithelial cells characteristic of epididymal secretory proteins. Ep = epithelium, Sp = luminal spermatozoa, Int = interstitial tissue. *D–F*,, Transmission immunoelectron microscopy was used to assess the localization of anti-MFGE8 antibodies in caput epididymosomes *in situ*,. These sections were labeled with appropriate secondary antibody conjugated to 10 nm gold particles, with (*F*,) controls including equivalent sections in which the primary antibody was omitted (*i.e.*, secondary antibody only). (*E*,) Depicts an enlargement of the boxed area in panel (*D*,). *G*,, The efficacy of biotinylated protein transfer into the post-acrosomal sheath of the head of caput spermatozoa (inset) was assessed following pre-incubation of caput epididymosomes with either anti-MFGE8 or anti-GAPDH antibodies, the latter being a protein that one would not expect to be associated with the membrane of extracellular vesicles. All analyses were performed in triplicate with representative immunofluorescence and immunogold images being shown. (*G*,) A minimum of 100 spermatozoa were assessed/replicate within each treatment group and graphical data are presented as means ± S.E.; * *p*, < 0.05.

Our collective data supporting the ability of epididymosomes to act as vehicles for modification of the maturing sperm proteome prompted us to seek a more detailed characterization of the mechanistic basis of this interaction. For this purpose, we elected to focus on MFGE8, a prevalent extracellular vesicle marker that has been variously implicated in the formation, secretion and uptake of exosomes from numerous cell types (59). Moreover, MFGE8 holds functional significance in terms of promoting sperm maturation owing to its downstream role in mediating initial adhesion to the egg coat (60). Consistent with previous evidence that mouse spermatozoa acquire MFGE8 during two distinct phases of their development; the first coinciding with spermatogenic development in the testis and the second attributed to uptake of the protein from secretions of the proximal epididymal segments, we documented discrete patterns of MFGE8 localization in testicular spermatozoa *versus*, those isolated from the distal caput epididymis. Thus, MFGE8 localized to the peri-nuclear domain of the head and the principal-piece of the flagellum in immature testicular spermatozoa (Fig. 8*A*,). In contrast, caput spermatozoa were characterized by additional foci of labeling within the post-acrosomal sheath and the anterior dorsal aspect of the head (Fig. 8*B*,). Equivalent labeling of caput epididymal tissue sections revealed intense MFGE8 localization within the supranuclear domain of principal cells and juxtaposed with spermatozoa within the lumen of the tract (Fig. 8*C*,); commensurate with that expected of a secretory protein. Accordingly, transmission immunoelectron microscopy analyses confirmed the presence of MFGE8 within epididymosomes, illustrating that immunogold particles were primarily restricted to the membrane of these vesicles and extended into stalk-like projections that formed at sites of interaction with the post-acrosomal sheath of spermatozoa (Fig. 8*D*,, 8*E*,). Notably, we were also able to demonstrate that pre-incubation of epididymosomes with anti-MFGE8 antibodies significantly compromised the efficacy of biotinylated protein cargo transfer between caput epididymosomes and spermatozoa (Fig. 8*G*,; *p*, < 0.05).

## DISCUSSION

A salient feature of the mammalian epididymis is its tremendous capacity for protein synthesis and secretion (12). Such activity underpins the primary roles of this tissue in promoting the functional maturation of the male gamete as well as their prolonged storage in a viable state (10, 20). Both roles necessitate efficient mechanism(s) of delivering protein, and presumably other regulatory cargo (50), to the developing sperm cells. Among the potential mechanisms that could facilitate such bulk transfer, there is now compelling evidence supporting at least two; namely, via nonpathological amyloid matrices (61) and/or epididymosomes (21). The former of these, which may also equate to epididymal dense bodies (62, 63), have been proposed to coordinate interactions between the epididymal luminal contents and spermatozoa, although the extent and biological significance of such interactions remain to be fully resolved. By contrast, the constitutive shedding of epididymosomes appears pivotal in terms of modulating sperm function (29). Indeed, our study adds to a growing body of evidence that, despite the relatively simple structure of these nano-sized membranous vesicles, they encapsulate an extremely rich and diverse proteomic cargo; and one that is commensurate with their putative role as key intermediaries in soma-spermatozoa communication (21).

Although the preparation of epididymosomes has been reported in several species, to date the comprehensive molecular profiling of their cargo has predominantly been restricted to that of large domestic species (*e.g.*, bull). Such species hold obvious advantages in terms of permitting the collection of large volumes of uncontaminated intraluminal fluids from along the length of the epididymal tract (21, 26). Regrettably, the application of equivalent collection protocols in smaller laboratory animals such as the mouse is technically very challenging, particularly in the context of recovering enough material to permit detailed end point characterization of the epididymosome proteome. Through necessity, we have instead pursued the isolation of epididymosomes from samples of luminal fluid obtained by puncture of the epididymis and processing by successive centrifugations to purify these vesicles. Although our previous studies have reported the utility of this approach in effectively eliminating cellular debris and sperm fragments (34), we readily acknowledge that we cannot entirely mitigate against the possibility of some epithelial and/or sperm cell contamination. One potential source of contamination is that of the cytoplasmic droplet, a nascent structure formed as a legacy of spermiogenesis during which spermatozoa are remodeled to remove most of their cytoplasm. This residual body is subsequently shed from the maturing sperm cell as they are conveyed through the epididymis. Of relevance to our study, the cytoplasmic droplet does contain numerous vesicles of roughly equivalent size to epididymosomes and could thus be co-isolated alongside this population of exosomes. Adding to this concern is that recognition that the cytoplasmic droplet serves to compartmentalize proteins implicated in membrane trafficking pathways, glucose transport, glycolysis, actin, tubulin and the proteasomal complex (64). Given this possibility for contamination, it was particularly reassuring that “extracellular exosome” featured among the top ranked cellular component categories identified in the epididymosome reported herein. Indeed, ∼82% of the total epididymosome protein cargo identified herein have previously been identified as genuine exosome-borne cargo in other cellular models. Similarly, “transport” and the ancillary categories of “protein transport” and “vesicle-mediated transport” were identified among the dominant biological processes annotated from the complete mouse epididymosome proteome. Moreover, immunoblotting confirmed that the epididymosome fractions studied herein were devoid of several sperm-specific markers, including well characterized proteins of testicular origin (*i.e.*, IZUMO1, ADAM3 and ODF2).

Although such evidence affirms our enrichment of epididymosomes, our compiled proteomic inventory did contain several categories of protein that one may not reasonably expect to be associated with an extracellular vesicle destined to be delivered to spermatozoa, or downstream segments of the male/female reproductive tracts. We did not anticipate the presence of proteins mapping to the broad functional categories of nucleotide binding and processing; with relatively large numbers of histone variants and ribonucleoproteins serving as cases in point. Although it is difficult to envisage the functional significance of such findings, they are certainly not without precedent. In this context, equivalent proteins have been documented in populations of exosomes originating from cell types as diverse as fibroblasts, mast cells, neural stem cells, dendritic cells, and oligodendrocytes (65–68). A subset of these proteins have also been recorded among the constituents of bovine epididymosomes (24). Further, it is acknowledged that mature spermatozoa do harbor the basic, and presumably obsolete, machinery to synthesize proteins, including numerous cytoplasmic and mitochondrial ribosomal proteins (69). It is widely held that such proteins simply represent remnants of the spermatogenic process. However, our study raises the intriguing prospect that the complement of these proteins may also be supplemented during post-testicular sperm development via interaction with epididymosomes. In the absence of evidence substantiating the synthesis of proteins from nuclear-encoded genes in sperm, such proteins may be subverted for alternative non-canonical functions or may be transmitted to the oocyte upon fertilization to participate in early embryogenesis. Further work is clearly required to substantiate these possibilities and thus refine our understanding of the biological implications of such enrichment.

Notably, functional annotation of the subset of the ∼18% of epididymosome proteins that were identified as not having not been reported in previous exosome proteomic catalogues, revealed an abundance of candidates linked to sperm maturation and/or fertilization; characteristics that one may logically expect to be associated with the functional transformation of the male gamete. Notable examples include the functional subunits of the chaperonin containing TCP1 complex (CCT/TRiC) as well as the putative interacting partners of ZP3R and ZPBP2, which have been implicated in the mediation of sperm-oocyte interactions (70, 71). Such findings suggest that this “non-conserved” subset of epididymosome-borne proteins may be of interest in helping to decipher the mechanisms driving the functional maturation of spermatozoa; potentially extending to a directed analysis of species-specific elements of these pathways. In a similar context, the abundance of these proteins assigned to the broad categories of “transport” and “oxidation-reduction” may hold important information in terms of dissecting the mechanistic basis of epididymosome-biogenesis/trafficking and protection of the mature gamete from free radical injury, respectively.

Our collective data also support the notion that epididymosome-sperm interaction is selective, with biotinylated epididymosome proteins being preferentially sequestered into a discrete physiological domain known as the post-acrosomal sheath, which is located within the posterior of the sperm head. Although such selectivity raises the prospect that unique compositional characteristics of the sperm plasma membrane directly influence the efficacy of epididymosome uptake, regrettably the mechanistic basis of this process has yet to be completely resolved. Notably, it has been argued that endocytic uptake, one of the principal routes of exosome internalization in somatic cells (33), is severely compromised in mature spermatozoa (72). Indeed, cytochemical investigations have reported that spermatozoa lack the machinery needed to internalize exogenous molecules via endocytosis and are also devoid of the lysosomal organelles that serve as the typical targets for endocytosed cargo (72). Further, mature spermatozoa are apparently also incapable of the active lipid recycling necessitated by endocytosis (73). In view of this evidence, it is possible that spermatozoa employ non-canonical pathways for uptake of epididymosomes, such as direct fusion occurring at the interface of the respective membranes (33). In this context, several complementary protein families implicated as key regulators of membrane/vesicle fusion-based pathways have been identified in spermatozoa (69) and in the epididymosome proteome reported herein. Examples of the latter proteins include those of the soluble NSF attachment protein receptor (SNARE) (*e.g.*, VAT1, STX3, STX4, STX5, STX6, STX7, STX8, STX12, STX16, STX17, STXBP2, STXBP3), RAB small GTPase (RAB1A, RAB1B, RAB2A, RAB2B, RAB3A, RAB5A, RAB5B, RAB5C, RAB6B, RAB7A, RAB8A, RAB8B, RAB9A, RAB11B, RAB13, RAB14, RAB18, RAB22A, RAB23, RAB24, RAB25, RAB35), and SEC (SEC11A, SEC13, SEC22B, SEC23A, SEC23B, SEC23IP, SEC24A, SEC31A) related families. Alternatively, it has been postulated that selective trafficking of epididymosome cargo to recipient sperm cells may be coordinated by the lipid raft-like properties of the vesicular membranes (74). In this regard, it is known that the lipid composition of mouse epididymosomes is dynamically remodeled in different epididymal segments such that these vesicles become progressively more rigid in the distal segments of the duct (75). At present it is not known what implications this has in terms of epididymosome-sperm and/or epididymosome-soma interactions. Clearly, additional work is needed to distinguish the relative contribution of the putative route(s) of sequestration of epididymosome contents into recipient cells. In guiding this work however, it is notable that previous studies have described the fusogenic properties of bovine epididymosomes and provided compelling evidence that such interactions lead to significant changes in the lipid and protein composition of epididymal sperm (22). These findings are consistent with our own immunoelectron microscopy analyses, which confirmed the presence of stalk-like projections forming at the interface of epididymosome-spermatozoa contact within the lumen of the epididymis. Such ultrastructural features have previously been recorded and taken as evidence of vesicle fusion between spermatozoa and oviductosomes (extracellular membrane vesicles released into the oviductal fluid) (76), raising the prospect of conserved mechanisms for facilitating cargo delivery between spermatozoa and the different populations of extracellular vesicles they encounter *en route*, to the site of fertilization. Accordingly, epididymosome protein transfer was significantly inhibited by antibody masking of MFGE8, a protein that possesses an RGD recognition motif implicated in integrin/ligand interactions that proceed cellular fusion (59).

In summary, the data obtained in the present study provides novel insights into the diversity of the proteomic landscape encapsulated within mouse epididymosomes. In accordance with previous work, our findings emphasize the fundamental importance of epididymosomes as key elements of the epididymal microenvironment necessary for coordinating post-testicular sperm maturation and storage. This work encourages further studies aimed at deciphering the biogenesis and cargo-sorting mechanisms responsible for epididymosome formation as well as more detailed examination of the mechanism(s) by which they can coordinate the delivery of proteinaceous cargo to recipient cells.

## DATA AVAILABILITY

The dataset (Dataset S1) analyzed here has been deposited in the Mass Spectrometry Interactive Virtual Environment (MassIVE) database with the dataset identifier MSV000082497 and is publicly accessible at: https://massive.ucsd.edu/ProteoSAFe/dataset.jsp?task=5cf36b642ed54e318f3b5dbb0d1db830. The FTP download is accessible via the link: ftp://massive.ucsd.edu/MSV000082497.

## Supplementary Material

Table S4

Table S1

Table S2

Table S3

Supplementary Figures

## Footnotes

^1^ The abbreviations used are:

EV: extracellular vesicle
ADAM3: a disintegrin and metallopeptidase domain 3 (cyritestin)
ADAM7: a disintegrin and metallopeptidase domain 7
B4GALT1: beta-1,4-galactosyltransferase 1
BWW: Biggers, Whitten, and Whittingham medium
CD: complement dependent
CUZD1: CUB and zona pellucida-like domain-containing protein
CLU: clusterin
DAVID: database for annotation, visualization and integrated discovery
DNM2: dynamin 2
DTYMK: deoxythymidylate kinase
FDR: false discovery rate
FLOT1: flotillin 1
GAPDH: glyceraldehyde-3-phosphate dehydrogenase
GLUL: glutamate-ammonia ligase (glutamine synthetase)
GO: gene ontology
HILIC: hydrophilic interaction chromatography
HSPA2: heat shock protein 2
HSP90B1: heat shock protein 90, beta (Grp94), member 1
IZUMO1: izumo sperm-egg fusion 1
LC-MS/MS: liquid chromatography-tandem mass spectrometry
MFGE8: milk fat globule-EGF factor 8 protein
MPC2: mitochondrial pyruvate carrier 2
NOLC1: nucleolar and coiled-body phosphoprotein 1
NUCB1: nucleobindin-1
ODF2: outer dense fiber protein 2
PDIA6: protein disulfide isomerase associated 6
PROM2: prominin 2
PSM: peptide spectrum matches
PSMD7: proteasome (prosome, macropain) 26S subunit non-ATPase, 7
SNARE: soluble NSF attachment protein receptor
TEAB: triethylammonium bicarbonate
TMT: tandem mass tag
ZP3R: zona pellucida 3 receptor
ZPBP2: zona pellucida binding protein 2.
