# Supplementary material for: Proteomic Profiling of Mouse Epididymosomes Reveals their Contributions to Post-testicular Sperm Maturation
Source: Mol Cell Proteomics. 2018 Sep 13;18(Suppl 1):S91–S108. doi: 10.1074/mcp.RA118.000946 (PMC6427233; doi:10.1074/mcp.RA118.000946)
Supplement: Supplementary Figures [file 139095_1_supp_192821_py5r6l.pdf]

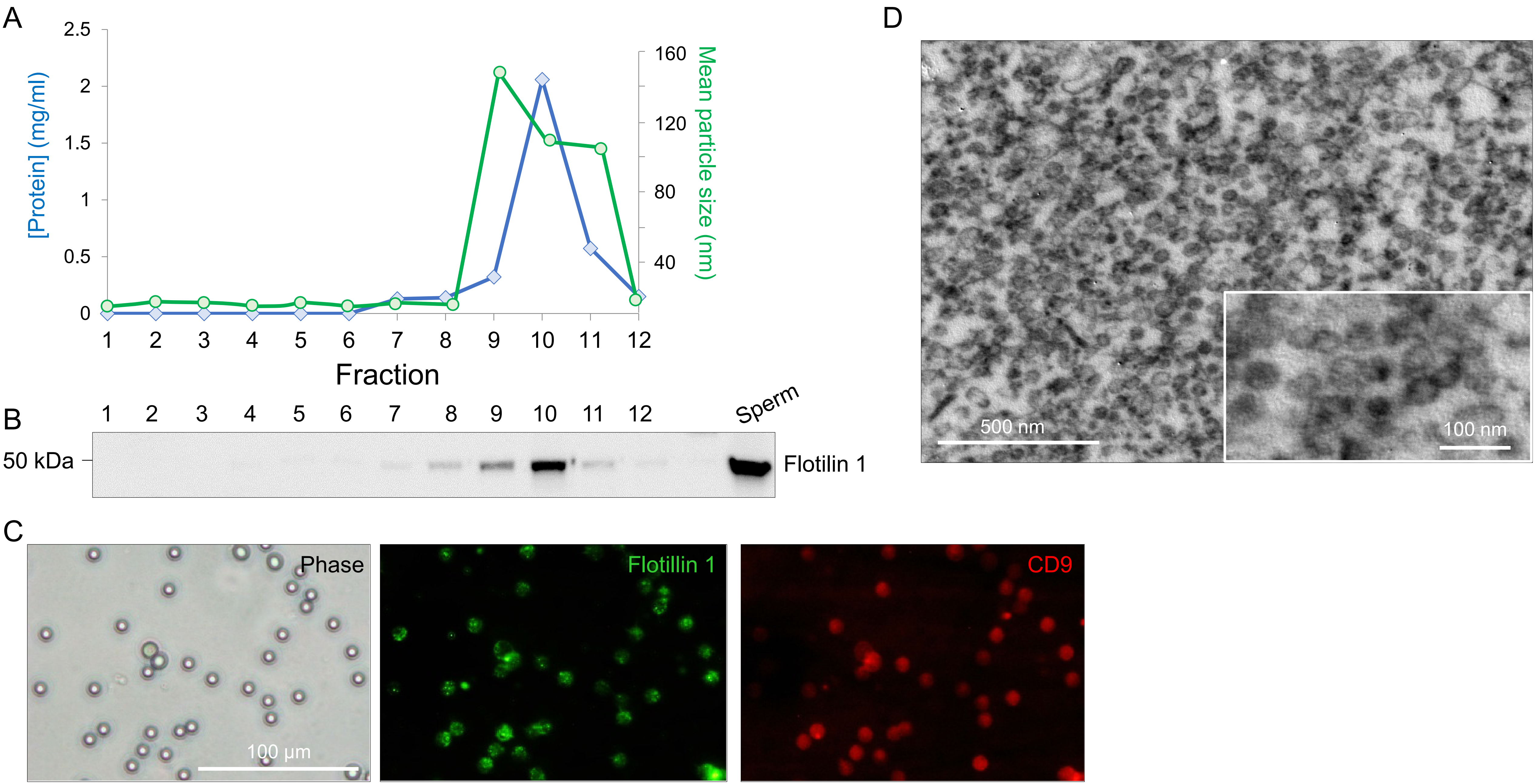
A
[Protein] (mg/ml)
Mean particle size (nm)
Fraction
B
Sperm
50 kDa
Flotilin 1
C
Phase
Flotillin 1
CD9
100 µm
D
500 nm
100 nm

Supplementary Figure S1: Assessment of epididymosome purity. The efficacy and purity of epididymosomes isolated for this study were assessed using criteria originally described in (34). Briefly, twelve equal fractions were recovered after ultracentrifugation over an iodixanol gradient and an aliquot of each prepared for (A) quantitative assessment of protein content (blue trace) and size heterogeneity (green trace). The latter was accomplished via measurement of mean particle size using dynamic light scattering. (B) Immunoblot analyses were performed to determine distribution of the exosome / epididymosome marker flotillin 1 (FLOT1) within each fraction. (C) A combination of FLOT1 and CD9 markers were also used to dual-label epididymosomes bound to aldehyde/sulphate latex beads (FLOT1 green, CD9 red). (D) Epididymosome preparations were also assessed via transmission electron microscopy to confirm the size and heterogeneity of the isolated populations. This experimental workflow was performed on all preparations of epididymosomes, irrespective of the downstream application.

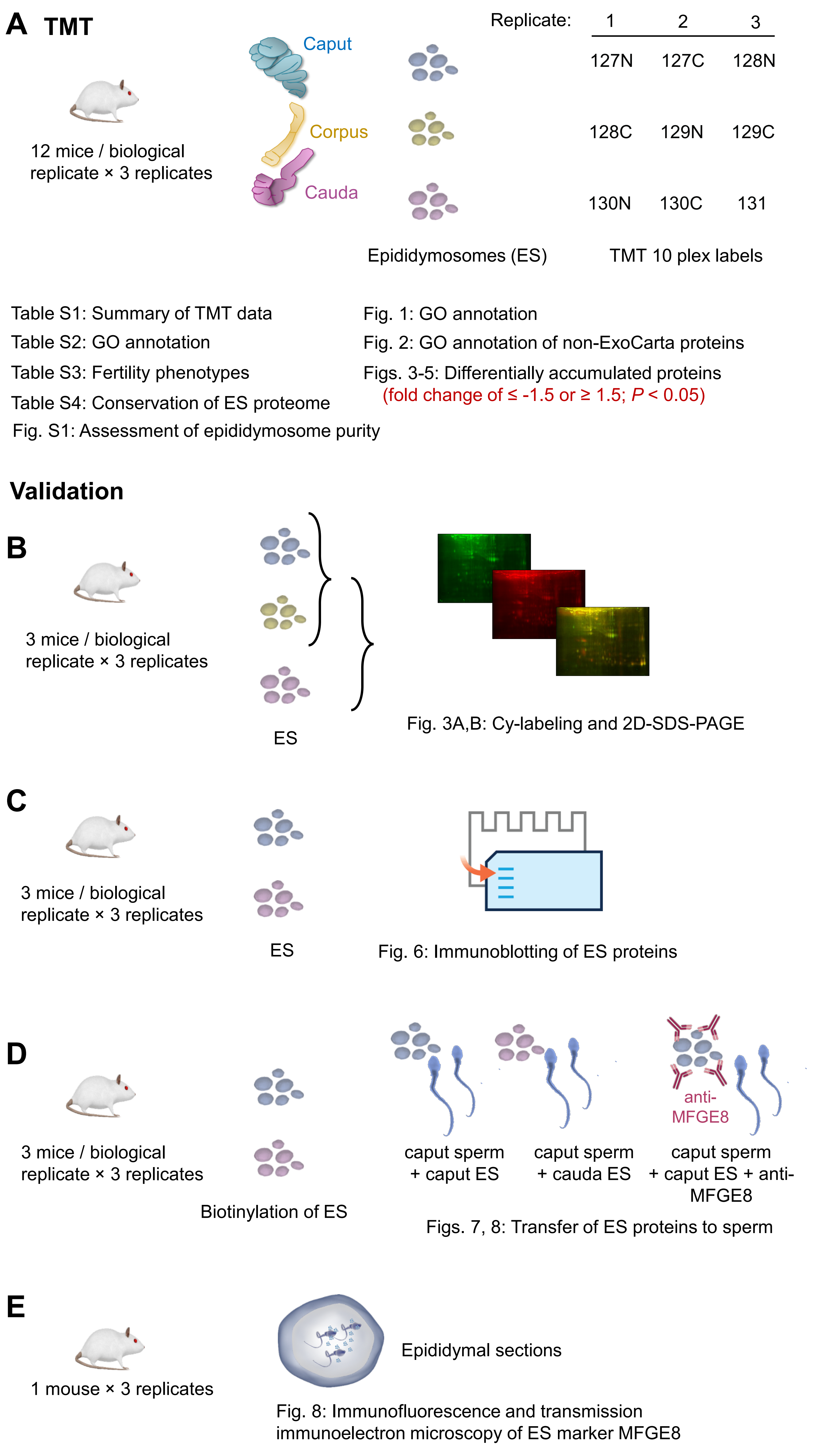
A TMT
Replicate: 1 2 3
Caput
127N 127C 128N
Corpus
128C 129N 129C
Cauda
130N 130C 131
12 mice / biological replicate × 3 replicates
Epididymosomes (ES)
TMT 10 plex labels
Table S1: Summary of TMT data
Table S2: GO annotation
Table S3: Fertility phenotypes
Table S4: Conservation of ES proteome
Fig. S1: Assessment of epididymosome purity
Fig. 1: GO annotation
Fig. 2: GO annotation of non-ExoCarta proteins
Figs. 3-5: Differentially accumulated proteins
(fold change of ≤ -1.5 or ≥ 1.5; P < 0.05)
Validation
B
3 mice / biological replicate × 3 replicates
ES
Fig. 3A,B: Cy-labeling and 2D-SDS-PAGE
C
3 mice / biological replicate × 3 replicates
ES
Fig. 6: Immunoblotting of ES proteins
D
3 mice / biological replicate × 3 replicates
Biotinylation of ES
anti-MFGE8
caput sperm + caput ES
caput sperm + cauda ES
caput sperm + caput ES + anti-MFGE8
Figs. 7, 8: Transfer of ES proteins to sperm
E
1 mouse × 3 replicates
Epididymal sections
Fig. 8: Immunofluorescence and transmission immunoelectron microscopy of ES marker MFGE8

Supplementary Figure S2: Experimental design. Biological replicates used in all experiments comprised pooled preparations of epididymosomes isolated from the appropriate epididymal segment (caput, corpus, cauda) of between three - twelve animals; with pooling necessitated on the basis of recovering sufficient protein for each downstream application. Three such biological replicates were used in each study. (A) Tandem mass tag (TMT) labeling was used in combination with NanoLC-MS/MS to generate a primary epididymosome proteomic inventory and facilitate comparative and quantitative proteomic analyses. Epididymosome proteins were identified as being differentially accumulated between epididymal segments if they experienced a fold change of $\leq$ -1.5 or $\geq$ 1.5; $P < 0.05$. (B - E) Validation of proteomic data was sought using a number of complementary strategies, including: (B) fluorescent Cy-dye labeling of epididymosome (ES) proteins and resolution via 2D SDS-PAGE; (C) immunoblotting of candidate epididymosome proteins was performed; (D) evaluation of the transfer of biotinylated ES proteins to spermatozoa; and (E) immunofluorescence and transmission immunoelectron microscopy of the ES marker MFGE8.
